# Recent Advances in Sugarcane Leaf Scald Disease: Pathogenic Insights and Sustainable Management Approaches

**DOI:** 10.3390/plants14040508

**Published:** 2025-02-07

**Authors:** Chun-Yan Kong, Kamal Priyananda Wickramasinghe, Chao-Hua Xu, Jun Mao, Hong-Bo Liu, Tanweer Kumar, Xiu-Qin Lin, Xu-Juan Li, Chun-Yan Tian, Pei-Fang Zhao, Xin Lu

**Affiliations:** 1 National Key Laboratory for Tropical Crop Breeding, Sugarcane Research Institute, Yunnan Academy of Agricultural Sciences, Yunnan Key Laboratory of Sugarcane Genetic Improvement, Kaiyuan 661699, China; wickramasinghekp@gmail.com (K.P.W.); xuchaohua_0435@sina.com (C.-H.X.); mj_raincat@163.com (J.M.); liuhongbo1982@126.com (H.-B.L.); tanweerkm_biologist@outlook.com (T.K.); linxiuqin07@163.com (X.-Q.L.); lixujuan2011@163.com (X.-J.L.); tianchy89@126.com (C.-Y.T.); hnzpf@163.com (P.-F.Z.); 2 Sugarcane Research Institute, Uda Walawe 70190, Sri Lanka; 3 Sugar Crops Research Institute, Agriculture, Fisheries and Co-Operative Department, Charsadda Road, Mardan 23210, Khyber Pakhtunkhwa, Pakistan

**Keywords:** sugarcane, leaf scald disease, *Xanthomonas albilineans*, host–pathogen interactions, genomic tools, pathogenicity

## Abstract

Sugarcane, a key cash crop in tropical and subtropical regions, is primarily cultivated for sucrose and bioethanol. However, Sugarcane Leaf Scald Disease, caused by the Gram-negative bacterium *Xanthomonas albilineans*, significantly threatens global sugarcane production. This review examines the disease cycle, epidemics, host–pathogen interactions, integrated management strategies, and future prospects for combating leaf scald. It highlights advancements in understanding pathogenicity, immune responses, and sustainable management of bacterial plant diseases to enhance control and prevention efforts. An analysis of GenBank data revealed 21 strains of *X. albilineans*, with some featuring complete genome maps and varying guanine-cytosine (GC) content. Advanced genomic tools, including clustered regularly interspaced short palindromic repeats (CRISPR), and molecular techniques, such as polymerase chain reaction (PCR), enable accurate pathogen detection and facilitate the identification of resistance genes, aiding breeding programs. Recent progress in whole-genome sequencing and reduced costs have enabled the assembly of multiple *X. albilineans* genomes, enhancing bioinformatics analysis. Despite these advancements, research on the global genetic diversity of *X. albilineans* remains limited. Addressing this gap is crucial for developing more sustainable strategies to manage leaf scald, ensuring stable sugarcane yields and supporting global production. Further studies will strengthen efforts to mitigate this significant agricultural challenge.

## 1. Introduction

Sugarcane (*Saccharum* spp. *hybrids*) is a perennial grass in the family Poaceae, contributing approximately 80% of the world’s total sugar production. It is the most efficient feedstock for bioethanol and biodiesel, accounting for about 40% of global biofuel production [1]. Sugar, a major agricultural product, is widely traded worldwide, and global sugar consumption has been steadily increasing. Thus, the production and development of sugarcane are crucial for ensuring sugar supplies and meeting human nutritional demands [2,3]. In addition, sugarcane, a C4 grass, is among the most efficient plants for assimilating CO₂ and light into sucrose, which contributes significantly to global sugar production. It is the only plant known to store high concentrations of sugar in its parenchyma and is considered a prime renewable energy crop due to its high biomass yield [4,5]. In tropical climates, sugarcane can achieve a high biomass production of 150–300 tons per hectare annually, depending on local conditions and management [6]. Additionally, by-products of sugarcane, such as straw and bagasse (fiber), can be used to produce cellulosic ethanol, a second-generation biofuel. Other products derived from sugarcane include molasses, rum, and cachaça (a Brazilian alcohol), while the plant itself can serve as thatch and livestock fodder [1].

As a tropical and subtropical herbaceous plant, sugarcane consists of five parts: roots, stems, leaves, flowers, and seeds, with a typical growth period of 9–10 months. In favorable conditions of light and temperature, sugarcane can be harvested once annually [7]. Unlike most crops, sugarcane is cultivated through stem segment propagation, a slower, asexual method that risks accumulating viruses and pathogens over time, causing degeneration of the germplasm, growth inhibition, and diseases, ultimately reducing yield and quality and constraining the industry’s development [8]. Currently, sugarcane is cultivated in over 121 countries, covering 27 million hectares worldwide, with over 130 known sugarcane diseases reported [1,8].

Leaf scald disease, caused by *Xanthomonas albilineans* (Ashby) Dowson, is a Gram-negative bacterium and a devastating threat to sugarcane [9,10], ranking among the top three bacterial diseases [11]. The disease manifests in three stages: the latent stage, characterized by the absence of external symptoms; the chronic stage, marked by multiple symptoms such as white streaks, chlorosis, necrosis, and shoot death; and, finally, the acute stage, characterized by leaf scald [12]. It was first reported in the Fiji Islands in 1908 and then in Australia in 1911; the disease has since been identified in 66 countries and different regions worldwide, including major sugarcane producers such as Brazil, India, China, and Thailand [13,14]. In China, leaf scald was first reported in the 1980s in Taiwan, Guangdong, and Fujian, based on symptomatology, cytological, and biochemical methods [15,16,17]. In 2007, *X. albilineans* was listed in the ‘Catalogue of Quarantine Pests for Import Plants to the People’s Republic of China’ and remains a primary target for quarantine of imported sugarcane germplasm [18]. Recently, the disease has been reported in sugarcane growing areas such as Guangxi, Yunnan, Hainan, and Zhejiang, and it is expected to spread in other sugarcane-producing regions of China [19,20,21,22,23].

The pathogen can remain asymptomatic in affected plants for a considerable length of time, and outbreaks can occur suddenly when environmental conditions are favorable, especially in sugarcane varieties that lack resistance to *X*. *albilineans* [24,25]. During this asymptomatic period, the pathogen is undetectable, as there are no visible external symptoms, making it difficult to identify and contributing to its global spread during germplasm exchange [26]. Susceptible sugarcane varieties have reported yield losses exceeding 40% and a severe reduction of 30% in juice quality [12,25,27]. To raise awareness about this severe disease, the present review article provides an overview of the research progress on the disease cycle, disease epidemics, pathogen characteristics, host–pathogen interactions, integrated disease management, and strategies for integrated disease control and prevention. Our aim is to provide important references for the study of the green control and prevention of sugarcane leaf scald and the mechanisms of pathogen–sugarcane interactions.

## 2. The Disease Cycle of Sugarcane Leaf Scald Disease

**Figure 1:**
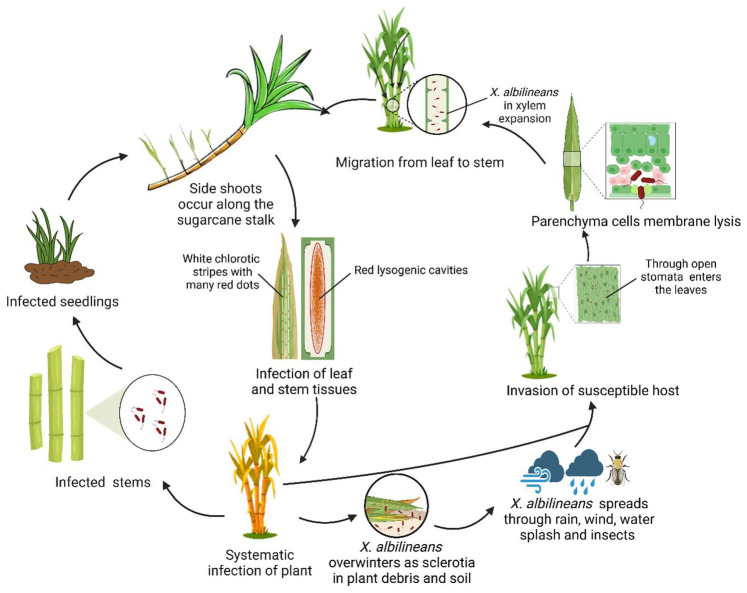
Schematic illustration of the life cycle of *X. albilineans*. *X. albilineans* can survive in plant debris in the soil and can colonize cane stems. It spreads from infected plants to healthy ones through various environmental and mechanical means. After the germination of colonized cane stems, the seedlings become infected. *X. albilineans* enter the plant through open stomata or wounds and spread systemically through the vascular system, leading to systemic infection. This infection manifests as white pencil-like lines on the leaves. As the infection progresses, chlorosis and albinism develop in the leaves of lateral buds, which is a typical characteristic of the disease. Information sources: [12,13,29,31,32,33,34,35,36,37,38,39].

**Figure 2:**
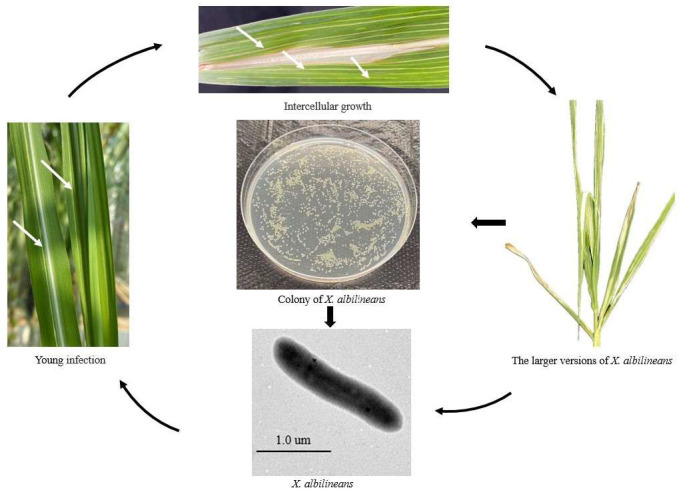
The disease cycle of sugarcane leaf scald. White arrows indicate the white pencil-like lines on the leaves that appear following infection by *Xanthomonas albilineans*.

The primary mode of transmission for *X. albilineans* is through infected stems, although weeds and infected plant debris also been reported to serve as potential sources [28,29]. Initially, the bacteria grow epiphytically on the leaf surfaces, aided by wind, rain, and insects. Later, they subsequently enter the host through open stomata or wounds, spreading systemically through the vascular system, and colonize the mesophyll parenchyma [24,30,31,32]. After infestation, the cell membranes of the thin-walled tissues of the leaves are cleaved, and xylem growth is inhibited. *X. albilineans* then migrates from the leaves to the stalks, affecting the tillering of the stems. Side shoots show faded white chlorotic stripes, which often become reddish over time, with the edges widening and accompanied by the appearance of numerous red dots. This progression is followed by necrosis and wilting of the infected leaves, eventually leading to plant death [12,28,29,31,32,33]. A schematic model illustration of the life cycle of *X. albilineans* is presented in Figure 1. At this stage, the mature *X. albilineans* adhere to the surface of the host tissue, completing the infestation cycle [28,29,31,32,33]. The disease cycle of sugarcane leaf scald is illustrated in Figure 2.

**Figure 3:**
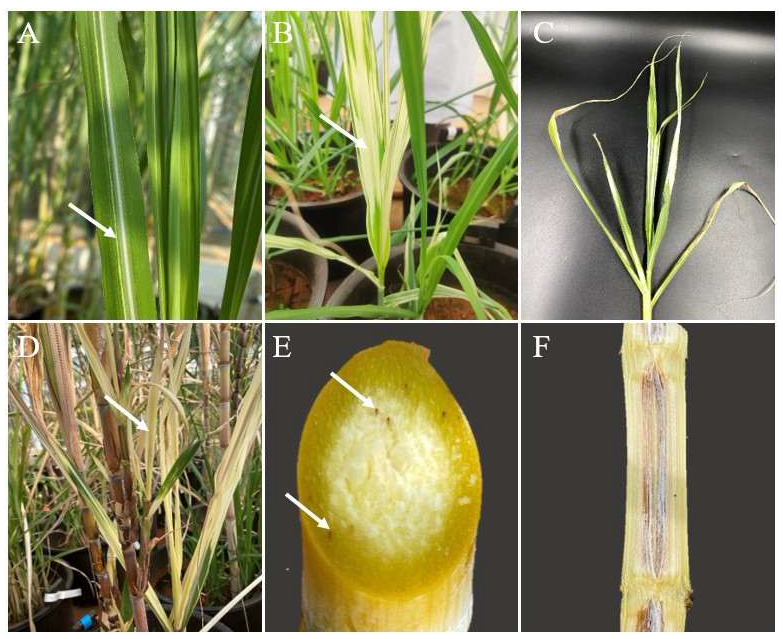
Typical symptoms of sugarcane leaf scald at different stages of infection. (**A**–**C**): early, mid-to-late, and late infection phases, respectively. (**A**) White pencil-like lines appear on leaves, as indicated by the white arrow. (**B**) Widespread discoloration and whitening of leaves, highlighted by the white arrow. (**C**) Leaf necrosis and wilting. (**D**) Chlorosis and albinism in the leaves of lateral buds, as indicated by the white arrow. (**E**) Node vascular bundles of a mature stem turn red, highlighted by white arrows. (**F**) Longitudinal section of an infected mature stem.

Typical symptoms of sugarcane leaf scald at different stages of infection in live specimens are shown in Figure 3. In the initial phase of the disease, *X. albilineans* causes the appearance of white to yellow chlorotic stripes that may be thin, pencil-like lines and parallel to the leaf veins, with neat edges [13,28,29]. Extensive fading and emerging leaves may also exhibit significant white chlorosis, resembling bleaching (Figure 3A,B). As the disease progresses, these leaf stripes and chlorosis turn into necrosis, causing the leaves to dry out and wilt. The extremities of the leaves curl inwards, giving the plant a scalded appearance (Figure 3C). In mature diseased stalks, side shoots develop from node buds along the stalk, with basal side shoots being more developed than those higher up. These side shoots show faded white chlorotic stripes, similar to the whiteness on the leaf blades of the main stems (Figure 3D). Infected stalks exhibit red node vascular bundles (Figure 3E), and longitudinal sections of the lower mature nodes of the cane stems in late-stage plants can reveal necrotic red lysogenic cavities within the cane stems (Figure 3F) [12,13,29,31,32,33,35,37,39].

## 3. Influence of Environmental Factors on Sugarcane Leaf Scald

The occurrence of plant-infesting diseases is tightly linked to the disease triad, consisting of host plants, pathogens, and environmental conditions. The interplay among these three elements determines the incidence and prevalence of the disease [40]. The host plant serves as the primary site for the disease, with resistant hosts mitigating and reducing pathogen accumulation. Conversely, susceptible hosts are more vulnerable, which is a fundamental prerequisite for disease incidence and epidemiology [29,40]. Previous reports indicate that different sugarcane varieties are susceptible to sugarcane leaf scald with varying degrees. Most of the sugarcane varieties in current production show susceptibility to sugarcane leaf scald, especially in areas with favorable climatic conditions for the disease [27,39]. Cervantes-Romero et al. [41] cited that the spread of leaf scald disease occurs in regions where temperatures are close to 30 °C and relative humidity exceeds 80%.

Sugarcane leaf scald is a highly transmissible bacterial seed-borne disease that spreads locally, primarily due to the use of infected cuttings from symptomless plants as seed stems [33]. At harvest, the pathogen can be transmitted to healthy plants via harvesting tools [20]. Additionally, the pathogen can be transmitted through various means such as soil [28], air [42], rain [43], sugarcane inflorescences [44], and human and animal activities [45,46]. In the absence of stringent isolation and quarantine measures and sensitive molecular detection techniques, pathogens can readily spread across different countries and regions through transfers and introductions [29,33].

The disease incidence and prevalence are influenced by the resistance of the sugarcane variety, the pathogenicity of the pathogen, environmental conditions in the cane field, and cultivation management practices [29,33]. The disease is more likely to occur when infected plants are exposed to environmental stresses such as drought or nutrition deficiency [29,33]. Previous studies have shown that the high incidence of leaf scald in sugarcane in Louisiana, USA, was associated with the area near the Gulf of Mexico, where a severe hurricane occurred in 1992 [27]. The disease incidence and epidemiology were aggravated by heavy rainfall or low temperatures during the hurricane period [27]. In Guadeloupe, France, the disease was found to be airborne, with pathogens being secreted through the drainage holes of sugarcane leaves and spreading through the air [28].

The number of *X. albilineans* and the severity of necrosis in sugarcane leaves are highly correlated with local precipitation, particularly during tropical storms [33,43]. Normal rainy season conditions are a primary cause of leaf scald pathogens spreading between sugarcane fields, with the distance between infected and healthy fields also affecting the degree of contamination [24,33,43]. According to Cervantes-Romero et al. [41], the symptoms of leaf scald differ between the chronic and acute forms of the disease and are influenced by weather conditions. Nicolau et al. [47] further highlighted that leaf scald hampers productivity, with water deficiency accelerating its progression. Taken together, these studies suggest that environmental factors, especially temperature and humidity, significantly impact the occurrence and prevalence of leaf scald in sugarcane fields in various regions, including the United States, France, Brazil, and other sugarcane growing areas around the world.

## 4. Research Progresses on Pathogenic Bacteria

### 4.1. Biological Properties of Sugarcane Leaf Scald Pathogens

*X. albilineans* belongs to the Bacteria, Proteobacteria, Gammaproteobacteria, Xanthomonadales, Xanthomonadaceae, Xanthomonas [48]. *Xanthomonas* has the following morphological characteristics: a Gram-negative bacterium, the colony morphology is round, light yellow, or honey yellow in color, with neat edges, elevated in the middle, with no mobility [49] (Figure 2). The bacterium is elongated and rod-shaped, with a size generally ranging from 0.4~0.7 µm to 0.7~1.8 µm [49]. This bacterium is an obligate parasitic, aerobic in nature, with an optimal growth temperature of 25~28 °C and optimal pH of 6.8~7.0, and slow growth in artificial medium, usually at least 4–6 days [36,37,45,49]. However, growth of *X. albilineans* requires amino acids, and it is antibiotic resistant [32]. Its biochemical properties include heptagenic hydrolysis, being negative for milk decomposition, inability to utilize ammonium salts, using nitrate or asparagine as a source of nitrogen for growth, nitrate not being reduced to nitrite, producing invertase but not urease, requiring methionine for growth, and being antibiotic resistant [45,49].

### 4.2. Genomic Characterization of the Sugarcane Leaf Scald

With the development of whole-genome sequencing technology and the reduction in sequencing costs, more and more *X. albilineans* genomes have been sequenced and completely assembled, providing an adequate database for bioinformatics analysis [50,51,52]. From the GenBank database, about 21 *X. albilineans* strains from different parts of the world were found to have bacterial genome information. These 21 *X. albilineans* strains include those from São Paulo (Brazil), strains Xa04, Xa11, Xa21, and Xa26; from Guadeloupe (France), strains GPEPC73, GPEPC17, and GPEPC86; from Reunion (France), strains REU17, and GPEPC86; strains REU174 and REU209 from Reunion Island; strain MTQ032 from Martinique; strains XaFL07-1, USA048, and Xa23R1 from Florida, USA; strains FIJ080 and CFBP2523 from Fiji; strain GAB226 from Gabon; strains HVO005 and HVO082 from Burkina Faso; strain PNG130 from Papua New Guinea; strain LKA070 from Sri Lanka; and strain Xa-FJ1 from China (Table 1).

The genomes of GPEPC73, Xa04, Xa11, Xa21, Xa26, and Xa-FJ1 strains had complete maps, while the genomes of the remaining 15 strains had only draft or partial maps. The genomes of these 21 strains varied in guanine and cytosine (GC) content from 62.80% to 63.30%. The genome of the GPEPC73 strain was composed of a circular chromosome with a size of 3.7687 Mb and three plasmids with sizes of 24837, 27212, and 31555 bp, containing 3310 genes and 3121 annotated proteins [50]. The chromosome of the GPEPC73 strain has a GC shift pattern typical of prokaryotic genomes, with two significant transitions near the start of the leading strand and the end of replication [50]. The complete genome of strain Xa-FJ1 consists of a circular chromosome of 3724581 bp and a plasmid of 31536 bp, containing 3259 genes and 3159 annotated proteins [51] (Table 1). The genomes of strains Xa04, Xa11, Xa21, and Xa26 contain 3188, 3312, 3365, and 3316 genes and 3141 and 3141, 3365, and 3316 annotated proteins, respectively [52] (Table 1).

### 4.3. Rapid Detection Methods of Pathogens

One of the key measures to prevent and control sugarcane leaf scald is the accurate detection of the pathogen and the rapid diagnosis of the disease [20]. The traditional isolation and culture methods are accurate and intuitive, as well as time-consuming [29]. Therefore, it is crucial to explore methods for rapid detection of the sugarcane leaf scald disease. Currently, the commonly used methods for rapid detection of the disease are Polymerase Chain Reaction (PCR), Restriction Fragment Length Polymorphism (RELP), Random Amplified Polymorphic DNA (RAPD), and Recombinase Polymerase Amplification (RPA), respectively [54,55]. The use of PCR can amplify DNA fragments specific to plant pathogenic bacteria, thus enabling rapid molecular detection of pathogenic microorganisms. Compared with traditional methods, PCR detection does not require tissue isolation and culture, and has the advantages of high specificity, rapidity, and sensitivity [14]. Pan et al. [56,57] designed two sets of specific primers, Ala4/L1 and PGBL1/PGBL2, for the detection of leaf scald in sugarcane. Wang et al. [58] designed a pair of specific primers XAF1/XAR1 to accurately detect *X. albilineans* based on the adenosine triphosphate-binding cassette (ABC) transporter protein gene *XALc_1791* of *X. albilineans*. Garces et al. [59] developed Taqman probes and primers targeting the *X. albilineans* biosynthesis gene cluster to establish a qPCR assay, and thus this method significantly improved detection sensitivity (Table 2).

## 5. Host–Pathogen Interactions

### 5.1. Genetic Diversity and Pathogenic Variation in X. albilineans

A growing body of evidence demonstrates the genetic plasticity among *X. albilineans* strains globally, as well as variations in pathogenicity. Earlier, a study by Persley [60] inoculated seven sugarcane varieties with isolates of *X. albilineans* from various sugarcane regions in Australia, revealing a diversity of pathogenicity among the strains. Similarly, Champoiseau et al. [61] and Huerta-Lara et al. [62] reported pathogenic diversity among *X. albilineans* isolates from the sugarcane regions of Guadeloupe (France) and Mexico, respectively. Rott et al. [63] prepared antibodies against isolates of *X. albilineans* from three geographical sources: Reunion Island, Burkina Faso, and Guadeloupe. Using lysophage and indirect immunofluorescence techniques, they studied the pathogens and found that 28 isolates from 11 countries could be classified into six different lysophage types and three different serological types. Rott et al. [64] further classified 215 *X. albilineans* isolates from 28 different countries or regions into three different serological types: serotype I, the most widely distributed, was found in strains from Australia, the USA, Guadeloupe (France), India, Mauritania, and South Africa; serotype II was found in strains from Africa; and serotype III was found in strains from Fiji, Sri Lanka, and the Caribbean Islands. Serotype IV, which was the most widely distributed, was found in populations of strains from Africa. In addition, Alvarez et al. [65] confirmed the serological diversity of the pathogen using monoclonal antibodies and DNA fingerprinting of 38 *X. albilineans* strains from various geographical locations. Similarly, Davis et al. [66] used genomic DNA restriction endonuclease polymorphism and pulsed-field gel electrophoresis (PFGE) profiling to identify 54 *Xanthomonas* glyptostroboides haplotypes and eight PFGE clusters (A–H). Pieretti et al. [67] reported ten PFGE clusters (A–J) globally, with most pathogenic strains associated with new leaf scald outbreaks classified under group B [14].

Molecular techniques for exploring the genetic diversity and genotyping of *X. albilineans* in sugarcane are crucial. Ntambo et al. [14] first reported the multi-locus sequence analysis (MLSA) approach based on the *gyrB*, *abc*, *rpoD*, *atpD*, and *glnA* genes to study genetic diversity and phylogeny among *X. albilineans* strains from China. These studies found a small degree of genetic variation in the four provinces of China, with strains belonging to the same group PFGE-B and showing high homology (from 99.5% to 100.0%) with the French strains GPEPC73, GPEPC17, GPEPC86, and MTQ032 and the American strain XaFLO7-1 [14,35]. Wu et al. [68] used sequence differences and phylogenetic tree analyses of housekeeping genes’ ATP-binding cassette transporters (ABC transporters), *atpD*, *gyrB*, and the *virB* gene of the type IV secretion system to classify 40 isolates into two types. The study showed significant differences in pathogenicity among the 40 strains, with only 5 strains displaying strong pathogenicity, 4 strains displaying medium pathogenicity, and 31 strains displaying weak pathogenicity. This research utilized MLSA and repetitive element sequence-based PCR (rep-PCR) to reveal the genetic diversity and population structure of *X. albilineans* strains in *Saccharum* spp. hybrids and *S. officinarum* from China, categorizing them into two phylogenetic groups (Group I and Group II) [69]. Unfortunately, limited studies on the genetic diversity of *X. albilineans* in sugarcane are reported worldwide, and further research is warranted to gain a more comprehensive understanding of this topic.

### 5.2. Mechanisms of Pathogenicity

*Xanthomonas* species, which are Gram-negative bacteria, are known for producing extracellular polysaccharide xanthan, contributing to the pathogenicity of *Xanthomonas* [29,70]. This characteristic is reflected in the taxonomic name of the genus, derived from the Greek word “xanthós”, meaning yellow, as these bacteria produce a yellow honey-like appearance on culture media [33]. *X. albilineans* exhibits unique pathogenic mechanisms, differing significantly from other *Xanthomonas*. Unlike other *Xanthomonas* spp., *X*. *albilineans* lacks the pathogenicity factors necessary for the growth and spread within host plants. Specifically, *X*. *albilineans* does not possess the Hrp-T3SS (a type of type III secretion system found in phytopathogenic bacteria that facilitates disease development in plants), but has a T3SS closely related to the *Salmonella* pathogenicity island 1 (*SPI-1*) gene, which injects effector proteins into a host cell’s cytoplasm or plasma membrane. This T3SS cluster is located near the terminus of the replication site and may have arisen through horizontal gene transfer [53,71]. Interestingly, the SPI-1 T3SS cluster is not correlated with plant colonization or virulence. It might be absent in at least one pathogenic strain of *X. albilineans*, and knockout mutants showed no impairment in promoting the disease [8,52]. The predicted effector region of the T3SS cluster is similar to the toxins predicted to interact with animals, suggesting that it may involve in association with potential insect vectors. This is particularly relevant when human-driven spread is not the primary means of propagation for this pathogen in sugarcane or other hosts [52,72]. Of note, the T3SS from *Salmonella* is predicted to have a shorter needle, as expected for bacteria–animal cell interactions, indicating its potential role in association with insect vectors when human-driven spread is not the primary means of propagation for the pathogen in sugarcane or other hosts [52,70]. In 1983, Birch and Patil [73] postulated the potential production of a diffusible phytotoxin during sugarcane infestation by *X. albilineans*. This hypothesis was confirmed in 1987, when it was established that the toxin was indeed produced by *X. albilineans* and was named albicidin. The molecule is relatively large and distinct, synthesized primarily through the action of the polyketide synthase (PKS), a non-ribosomal peptide synthetase (NRPS) gene cluster [74].

The *X. albilineans* toxin biosynthesis gene cluster has been fully cloned and sequenced, revealing 20 open reading frames (ORFs), including one PKS-NRPS gene (*albI*) and two *NRPS* genes (*albIX* and *albIV*), as well as several putative resistance regulator and modifier genes [75,76]. At the same time, albicidin is a potent DNA gyrase inhibitor that prevents DNA replication in bacteriophages, bacteria, and within plastids of plant cell [70]. Although, albicidin is widely accepted as the primary contributor to the leaf phenotype, it is not considered a virulence determinant, as the GPE PC73 strain produces symptoms without producing albicidin [70]. Recent evidence by Kortright et al. [77] has demonstrated the potential use of albicidin as an antibiotic, which may confer an advantage to the *X. albilineans* bacterial population within plant tissues by inhibiting the growth of endophytes. The *X. albilineans* exhibit albicidin activity in white streaks provides a competitive advantage over other bacteria during its colonization of sugarcane. This activity may prevent the differentiation of sugarcane chloroplasts, resulting in the characteristic of white chlorotic stripes symptom on the foliage [74,78,79]. Furthermore, deletion of the white-striped *Xanthomonas* toxin gene has been shown to attenuate the pathogen’s ability to exhibit latency or systemic infestation, a key factor for pathogenicity [74].

*X. albilineans* is a unique species within the genus *Xanthomonas*, with a genome size smaller than that of other *Xanthomonas* species (typically 5 Mb). *X. albilineans* strains possess 522 genes not conserved in other species of the genus *Xanthomonas*, along with the presence of two intermediate short palindromic repeats [50,67]. These unique genomic features have been demonstrated to positively impact on *X. albilineans* colonization in the xylem of sugarcane [10,50,51]. Analyses of Xa-FJ1 and GPE PC73 revealed associations between phage integration, homologous recombination, transposable elements, and CRISPR systems with 16 insertion/deletion fragments, resulting in the identification of 10 and 82 specific genes in Xa-FJ1 and GPE PC73, respectively. Some of these genes were linked to phage-associated proteins, zona pellucida toxins, and DNA methyltransferases [51].

Recent studies have presented the complete genome sequences of four Brazilian *X. albilineans* strains with varying levels of virulence and compared them to those of the GPEPC73 reference strain and FJ1. Based on the aggressiveness index, the strains were classified as highly aggressive (Xa04 and Xa11), intermediate (Xa26), and least aggressive (Xa21). Regarding genome structure, Xa04 shares most of its genomic features with Xa26, while Xa11 shares most of its features with Xa21. Furthermore, *X. albilineans* strains exhibit more CRISPR clusters and four additional prophage insertions compared to the previously sequenced GPEPC73 and FJ1 strains. Incorporating the aggressiveness index and in vitro cell biology into these genomic features suggests that disease establishment in *X. albilineans* is not determined by a single factor, as in other *Xanthomonas* species [52]. Comparative albicidin inhibition rings and in vitro growth curves of the four strains also do not correlate with pathogenicity, and leaf scald disease is not associated with a single shared characteristic between the most or the least pathogenic strains [52]. The pathogenesis of *X. albilineans* is complex, and recent studies on the function of the two-component system of *Xanthomonas* in pathogenesis have provided new ideas to investigate the pathogenesis of *X. albilineans*. The Xa-JG43 strain of *X. albilineans* was found to contain several two-component systems, among which the deletion of the *rpfg* gene in the *rpfc/rpfg* two-component system led to a decrease in the pathogenicity of the bacterium [38]. Recently, researchers produced a *phoq* knockout mutant in the Xa-JG43 strain using the homologous recombination method [80]. Compared with the wild-type strain Xa-JG43, the pathogenicity of the Xa-*phoq* knockout mutant was inhibited [80]. Although the determinants of host- or tissue-specificity of *X. albilineans* remain unclear, the presence of cell wall-degrading enzymes with specific characteristics in its genome may contribute to its ability to spread and cause pathogenicity in the xylem [8]. All cell wall-degrading enzymes of *X. albilineans* contain a cellulose-binding domain and a long linkage region. These enzymes can degrade cell wall components and disrupt sugarcane structural membranes, enabling the pathogen to utilize the degraded cell wall products to enhance its propagation within sugarcane xylem ducts [81,82].

Whole genome sequencing of *X. albilineans* has revealed several potential candidate genes associated with pathogenicity. These candidate genes include a pathogenicity regulator (*rpf*) involved in the biosynthesis of small diffusible signaling molecules, as well as a diffusible signaling factor (DSF) synthesized by the *rpfF* gene, which functions similarly to a long-chain fatty acyl cofactor [32,71]. The DSF regulates cell-to-cell communication, such as population sensing, and influences the expression of pathogenicity related genes. When the *rpfF* or *rpfC* gene (a hybrid two-component DSF sensor) is disrupted, it leads to reduced or absent virulence in strains of different *Xanthomonas* species, such as *Xanthomonas campestris* pv. *Campestris, Xanthomonas oryzae* pv. *oryzae*, and *Xanthomonas axonopodis* pv. *citri* [32,83]. However, in *X*. *albilineans* strain XaFL07-1, isolated from Florida, U.S.A., single or double mutations in the *rpfG* and *rpfC* genes resulted in even stronger infestation ability compared to the wild-type strain. Nevertheless, complete removal of *rpfG* and *rpfC* genes reduced the strain’s pathogenicity [84].

Additionally, the *albXXI* gene, encoding pancreatic acinar cell carboxyl esterase, along with four ABC transporter protein genes, three non-ribosomal peptide synthetase (NRPS) genes, one methyl-accepting chemotactic protein gene, and one oxidoreductase gene, were identified as potential contributors to its pathogenicity mechanism [67,76]. Researchers detected numerous pathogen–host interaction (PHI) genes and virulence factors within 17 genomic islands (GIS) of strain JG43, 6 of which were directly associated with pathogenicity [71].

With advances in whole-genome sequencing and the availability of extensive microbial genome data, strain typing has become more precise and accurate through core genome multilocus sequence typing (cgMLST). Core genome elements of *X*. *albilineans* include adhesion genes, cell wall-degrading enzymes, polysaccharide synthesis genes, genes involved in secretion systems, non-ribosomal peptide synthetase (NRPS), DSF-related genes, and the flagellar operon [52].

Sugarcane leaf scald, on the other hand, is characterized by acute and chronic symptoms or even acute onset after months of incubation due to factors such as the sugarcane growing environment, sugarcane variety, and concentration of pathogenic bacteria, proving the existence of a complex pathogenic mechanism that has not yet been clarified.

### 5.3. Sugarcane Immune Response Induced by Leaf Scald Pathogen

Plant–pathogen interactions are part of a complex process mediated by plant-derived molecules and pathogens, comprising proteins, sugars, and lipopolysaccharides [85]. These interactions involve two-way communication where the plant recognizes and defends itself against a potential pathogen and the pathogen manipulates the plant’s biology to create a suitable environment for its growth and reproduction. Recognition of pathogen or microbe-associated molecular patterns (PAMPs or MAMPs) is the first step in an active plant defense response, known as PAMP-triggered immunity (PTI). Recognition of specific effectors produced by the pathogen by the plant is the second step of strong plant defense known as effector-triggered immunity (ETI), which involves localized programmed cell death to halt pathogen growth [85]. In contrast, the pathogen suppresses various components of PTI by delivering effector proteins into the host plant’s cytoplasm [85,86]. In sugarcane leaf scald pathogen–sugarcane interactions, sugarcane senses the molecular patterns or effector proteins of the pathogen bacteria. Immune signals, such as pathogenesis-related (PR) or other resistance (R) proteins, are recognized and transmitted to activate defense-related genes and produce specific immune compounds, initiating immune responses [86,87]. Defense-related genes include those encoding protein kinases, transcription factors, catalase, and defense compounds like phenolic acids, flavonoids, and macromolecular compounds [32,38].

Sugarcane is an aneuploid and allopolyploid crop characterized by a high chromosome number and a large genome size. Analyzing its reference genome is not only critical for understanding the species’ genetic information, but also serves as a foundation for studying immune responses to leaf scald pathogens [1,2,8].

In 2017, a 400 Mb segment of the genome of the sugarcane cultivar SP80-3280 was released [88]. A year later, Zhang et al. [89] sequenced the complete genome of the tetraploid sugarcane cultivar AP85-441, derived from in vitro culture of octoploid SES-208 anther cuttings, yielding a 3.13 Gb assembly with 35,525 predicted genes. In 2021, the genomes of two *Miscanthus* species, *M. lutarioriparius* [90] and *M. floridulus* [91], were deciphered. Moreover, the autotetraploid *S. spontaneum* Np-X (2n = 4x = 40) was assembled, resulting in a 2.76 Gb genome with centromere structures mapped on over 92% of its chromosomes [92]. Shearman et al. [93] reported that the sugarcane cultivar Khon Kaen 3 (KK3) achieved a partial genome assembly at the chromosome level, consisting of 104,477 contigs totaling 7 Gb, scaffolded into 56 pseudochromosomes containing 5.2 Gb of sequences.

In 2023, the diploid *Erianthus fulvus* genome was assembled to the chromosome level [94,95]. By 2024, the genome of R570 was sequenced and assembled, constructing a 5.04 Gb genome [96]. Furthermore, in 2024, the genome of modern Sugarcane hybrid cultivar Zhongzhe1 was successfully deciphered at the chromosome level, achieving a breakthrough with complete genome assembly of 114 chromosomes [97]. These advancements create an invaluable platform for cloned gene and molecular marker development, enhancing studies on sugarcane’s immune response to leaf scald pathogen.

Interestingly, the brown rust resistance gene *Bru1* was identified in the sugarcane variety R570 using molecular markers closely linked to disease resistance. Scholars developed two key molecular markers, R12H16 and 9O20-F4, which are strongly associated with *Bru1* [98,99]. These markers have since been incorporated into PCR-based marker systems, facilitating molecular marker-assisted breeding in various sugarcane breeding programs worldwide [100,101,102]. Recent studies analyzing the R570 genome in detail not only revealed the contribution of the parental species to the genetic characterization of modern varieties, particularly regarding disease resistance, but also clarified the candidate gene for the brown rust resistance gene *Bru1*. This provides a molecular means for improving disease resistance in sugarcane [96]. The discovery and successful application of *Bru1* serve as a notable example of how molecular markers can enhance sugarcane breeding.

In related research, Australian scientists have made progress in combating sugarcane leaf scald disease. They isolated the biocontrol bacterium *Pantoea dispersa* from sugarcane leaves affected by leaf scald and subsequently cloned the detoxification gene *albD* from this bacterium. Overexpression of the *albD* gene in the sugarcane cultivar Q63 led to transgenic plants with significantly enhanced resistance to leaf scald, showcasing the potential of genetic interventions to improve disease resistance in sugarcane [45,103]. Gutierrez et al. [104] used 89 progenies from a cross between the sugarcane leaf scald-resistant variety LCP85-384 and the disease-susceptible variety L99-226 to construct a molecular map using 1948 simple repetitive sequences, coding region sequences, and single-nucleotide polymorphisms. The map identified eight quantitative trait loci (QTLs) associated with leaf scald resistance. These QTLs and molecular markers provide a basis for exploring the mechanism of leaf scald resistance and for molecular marker-assisted breeding.

Analyzing the transcriptional changes in sugarcane infested with leaf scald is crucial to understanding its immune mechanism. Comparative transcriptomic studies have found that plant–pathogen interactions, hormone signaling, and phenylpropanoid biosynthesis play significant roles in sugarcane’s response to leaf scald infestation [85,105]. Meng et al. [29] applied comparative proteomics analysis to identify 285 differentially expressed proteins in two sugarcane cultivars, LCP 85-384 (resistant) and ROC20 (susceptible), during infection with *X. albilineans*. These proteins were primarily enriched in pathways related to secondary metabolite biosynthesis, amino acid metabolism, phenylpropane biosynthesis, ubiquitin-mediated protein hydrolysis, and glutathione metabolism [29].

Several important functional proteins, such as mitogen-activated protein kinase (MAPK), non-specific lipid transport protein (nsLTP), and basic helix-loop-helix protein (bHLH), were identified at the genome-wide level and are closely related to sugarcane’s response to leaf scald infestation [106]. Studies show that the number of differentially expressed genes (DEGs) in LCP 85-384 was lower than in ROC20, suggesting less intense global gene expression in the resistant cultivar within the first 72 h after inoculation compared to the susceptible cultivar. Conversely, in SES208 (resistant cultivar) and LAPurple (susceptible cultivar), the resistant variety exhibited a much higher number of DEGs than the susceptible variety. This indicates that sugarcane leaf scald-induced immune response genes are mainly enriched in plant–pathogen interactions, glutathione metabolism, phytohormone signaling pathways, and phenylpropanoid biosynthesis. The number of DEGs related to leaf scald resistance is not directly related to resistance [29,87,107]. Additionally, genes such as ubiquitin-activating enzyme genes (*UBA1*), Argonaute (*AGO)* protein genes, *UDP* glycosyltransferase genes, *nsLTP* genes, TGACG motif-binding factor (*TGA*) genes, and *ShWRKY* genes are associated with sugarcane resistance to the disease [29,107]. Although the cloning of sugarcane leaf scald-related genes and the development of molecular markers have not yet been reported, research progress indicates that sugarcane’s immune response to leaf scald is a complex process regulated by multiple physiological and biochemical pathways. These pathways initiate early pathogen recognition and signal transduction, activate transcription factors and defense-related genes, and generate a series of defense responses that ultimately manifest as resistance to leaf scald.

The complex interaction between sugarcane and the leaf scald pathogen involves multiple physiological and biochemical pathways. Advances in molecular markers, QTL identification, and genetic engineering are paving the way for enhanced disease resistance and more efficient breeding strategies in sugarcane.

## 6. Integrated Disease Management (IDM) of Sugarcane Leaf Scald

As sugarcane is an asexually propagated crop, successive years of lodging lead to repeated infestations and accumulation of multiple pathogens in plants [29]. This accumulation triggers sugarcane diseases, resulting in reduced yield and sugar content, which have significant negative impacts on the development of the sugar industry around the globe [69,108,109]. To effectively manage sugarcane leaf scald, understanding the disease’s occurrence, characteristics, and influencing factors is crucial. Prevention and control efforts primarily focus on three aspects: agricultural control, chemical control, and biological control.

### 6.1. Agricultural Practices for Sugarcane Leaf Scald Disease Control

Agricultural control measures for sugarcane leaf scald consist of two main strategies: planting resistant sugarcane varieties and adopting scientific agronomic practices [39,110].

#### 6.1.1. Screening Methods and Breeding Materials for Resistance to Sugarcane Leaf Scald Disease

Employing disease-resistant genotypes has proven to be the safest and most cost-effective and efficient method for controlling the leaf scald disease [110]. Therefore, screening, evaluating, and utilizing disease-resistant parents or germplasm resources are critical for disease-resistant breeding, and disease resistance is routinely assessed [39,111]. Currently, sugarcane leaf scald inoculation identification methods primarily include the impregnated inoculation method, the leaf-cutting method, and the decapitation method [25,39,68,111]. Among these, the impregnated inoculation method is the most widely used both domestically and internationally for identifying sugarcane resistance to leaf scald. In this method, during the early stage of sugarcane elongation (about 5–6 nodes), the head is cut off at the auricle of the +1 leaf, and the portion of the sugarcane plant above the growing point is removed. A bacterial suspension is then dripped onto the cut surface, making the method suitable for determining the pathogenicity of the pathogen and evaluating sugarcane varieties’ resistance to leaf scald. This method holds significant practical value [25,39,59,78,112,113,114,115].

The leaf-cutting technique, used during the seedling stage of sugarcane (consisting of 3–5 leaves), involves using a sterile scalpel, dipping it into a suspension of *X. albilineans*, and then cutting off one-third of the leaf apex of each leaf. After inoculating each plant, the scalpel is re-dipped in the bacterial suspension. This method is quick and convenient, suitable for validating the Koch postulates of infestation disease pathogens [35,111]. Wu et al. [68] used the impregnated inoculation and decapitation methods to identify resistance to leaf scald in sugarcane. The study revealed that the impregnated inoculation method took a shorter time than the decapitation method and that the seedling stage was easier to observe compared to the elongation stage. However, the basis of investigation was not as stable as that of the decapitation method due to the inconsistent rate of seedling emergence [68].

Inoculation methods vary from region to region, and the degree of damage varies from host to host, resulting in different manifestation and grading criteria for sugarcane leaf scald [59,68,111,113]. Daugrois et al. [42] categorized the disease into four grades based on the severity of disease symptoms on the leaves, while Rott et al. [25] categorized it into five grades. Gutierrez et al. [115] meticulously categorized sugarcane leaf scald infestation into grades 0–9 based on the severity of the disease. The current study found that the most commonly used method is to categorize the disease into five grades based on its severity (Table 3) and then evaluate the resistance of sugarcane varieties to leaf scald based on disease indices (Table 4) [25,59,66,68,111,113,116].

Disease index calculation formula adopted from Rott et al. [113]:

$$Disease\;index=\frac{\sum (Number\;of\;incidence\;levels\times Number\;of\;plants\;in\;the\;corresponding\;class)}{Highest\;morbidity\;level\times Total\;number\;of\;plants\;surveyed}\times 100$$

At least one-hundred-and-twenty-eight sugarcane materials are known to be resistant to leaf scald, with sixty-nine from China, thirty-nine from the United States, nine from Australia, four from Mexico, four from France, two from Gabon, and one from Cuba (Table 5). According to the degree of resistance, there are 24 with high resistance, 63 with disease resistance, and 41 with medium resistance (Table 5), representing potential resources for studying the resistance mechanism of leaf scald and breeding resistant varieties.

Fu et al. [111] and Wei et al. [116] utilized the identification method to determine leaf scald resistance in the sugarcane variety Zhongzhe13. However, the results indicated resistance [116], while Fu et al. [111] revealed moderate resistance. These results suggest that the assessing resistance in the same variety using the same method may not be consistent. Therefore, comprehensive evaluations in the field, combining artificial inoculation tests with multi-year and multi-point trials, are necessary to achieve stable and dependable results.

#### 6.1.2. Agronomic Practices for the Integrated Management of Sugarcane Leaf Scald

Agronomic measures play a crucial role in the integrated management of sugarcane leaf scald. Several key aspects of scientific agronomic measures has been identified, including the following: (1) employing clean harvesting tools and removing diseased leaf stubs [8], (2) pre-soaking seed stems for 48 h in cold running water (15–25 °C) followed by a 3 h soak in hot water at 50 °C to reduce the number of pathogens carried by seed stems [33], (3) thoroughly fumigating and disinfecting soil before planting to eliminate pathogenic bacteria, particularly effective for soil-borne diseases [137], (4) controlling temperature and humidity and ensuring proper aeration and light by adjusting planting density [137], and (5) implementing crop rotation with non-host crops every 2–3 years to prevent the occurrence of sugarcane leaf scald [8].

### 6.2. Chemical Control of Sugarcane Leaf Scald Disease

Chemical agents are widely used due to high preventive effect, low cost, and other benefits. Previous studies have indicated that the use of “Kocide 2000 wettable powder” and “agro-neophytomycin sulfate”, when applied at appropriate concentrations, can effectively control the proliferation of the disease, particularly in the early stages [138]. Additionally, the application of inorganic silicon has been found to increase plant height and width, as well as regulate disease resistance through metabolic pathways related to hydrogen peroxide (H_2_O_2_), L-phenylalanine ammonia-lyase (PAL), salicylic acid (SA), and jasmonic acid (JA) [139]. Ethylicin significantly enhances the photosynthetic capacity of sugarcane leaves, improves root vigor, and boosts the activities of endogenous defense enzymes. It also increases the content of secondary metabolites, including total phenols and flavonoids, and osmotic regulators such as soluble sugars, soluble proteins, and proline. These effects help to reduce membrane lipid peroxidation damage and improve the resistance of sugarcane, thereby promoting plant growth [140].

The preventive efficacy of chemical agents such as inorganic copper and silicon, organic copper, and Ethylicin typically ranged from 36.2% to 81.4%. For chemical composite bactericides, a 20% thiediazole copper (Lonchococcus) suspension (1:500) combined with 6% Chunrexin wettable powder (1:250) achieved a control efficiency of 48.2% [141]. Additionally, 1000 mg/L Cu(OH)_2_ with 0.05% organosilicon additive yielded a control efficiency of 50.3% [142]. The control efficiencies of Mancozeb and Ethylicin in ratios of 2:3 and 1:1 were 85.9% and 81.6%, respectively, under field conditions [137]. These studies indicated that a single chemical agent or a combination with other biological agents can enhance control efficacy.

### 6.3. Biological Control

The prolonged use of chemical agents can lead to the development of pathogen resistance and environmental pollution. However, there are few reports on the biological control of leaf scald. Biological control, with its advantages of low residue, low toxicity, and renewability, has emerged as a key approach to integrated disease prevention and control [137]. This method mainly employs biological agents, such as antagonistic microorganisms and plant-derived antibiotics, to manage diseases. For instance, gluconacin, a bacteriocin obtained from *Gluconacetobacter diazotrophicus* PAL5, has been found to inhibit the growth of *X. albilineans* [37]. Previous studies have also shown that antagonistic bacteria with enhanced defense mechanisms, such as *Gluconacetobacter diazotrophicus*, a nitrogen-fixing endosymbiont of sugarcane, can inhibit the production of bacterial gum, thereby impeding the growth of *X. albilineans* [143]. Additionally, the exogenous application of Jasmonic acid (JA) and nano selenium (nano-Se) has been found to inhibit malondialdehyde (MDA) accumulation, reduce reactive oxygen species (ROS) and hydrogen peroxide (H_2_O_2_) levels, enhance the expression of JA pathway-related genes, and improve the activity of defense-related enzymes, ultimately increasing sugarcane’s resistance to *X. albilineans* [11,97,144].

## 7. Future Perspectives

Sugarcane leaf scald is a significant challenge in the most sugarcane-producing countries/regions in the world, primarily due to its latent infections and the lack of visible symptoms during the incubation period. Therefore, research on sugarcane leaf scald has primarily focused on the occurrence and prevalence of the disease and its comprehensive prevention and control. However, further research is needed on host–pathogen interactions, the pathogenic mechanism, the exploration of precise methods for resistance identification, and the sugarcane immune response, as well as comprehensive prevention and integrated disease management.

### 7.1. Future Perspectives on Host–Pathogen Interactions

Based on the current research progress regarding the interactions between pathogenic bacteria and host plants, several areas need further investigation. A more in-depth analysis of the key pathogenic genes and mechanisms of these bacteria is necessary. Secondly, a comprehensive analysis of the mechanism of sugarcane resistance to leaf scald is required, including early detection of resistance, exploration of disease resistance targets, and the application of these findings to the selection of sugarcane varieties. Previous studies have identified PR resistance proteins, WRKY proteins, mitogen-activated protein kinases, TGA transcription factors, *nsLTP* genes, antioxidant enzymes, and flavonoid defense metabolites as being associated with sugarcane leaf scald resistance.

However, further validation of these disease resistance functions is essential. Future research on sugarcane leaf scald should prioritize identifying resistant varieties. Notably, sugarcane is a highly heterozygous, asexually reproduced crop with a complex genetic background, making it challenging to achieve ideal trait combinations through traditional hybridization alone. However, advances in molecular biology and high-throughput gene technologies, including molecular marker-assisted selection and genetic engineering, have helped overcome limitations of traditional crossbreeding. Breeders should also leverage the complete genome sequencing of sugarcane autotetraploid *S. spontaneum* (AP85-441/Np-X), *Erianthus fulvus*, and the modern cultivated hybrid varieties Zhongzhe1 and R570 to better understand the genetic mechanisms underlying leaf scald resistance.

Advanced molecular techniques, such as genome-wide selection, transgenics, KASP (kompetitive allele-specific PCR), CRISPR/gene editing, synthetic biology, and artificial intelligence could be adopted to efficiently integrate desirable allelic variants into the sugarcane population resistant to leaf scald. Research on fine localization, gene cloning, and resistance mechanism against leaf scald is underway. These methods will accelerate breeding efforts, enabling the rapid and precise development of sugarcane varieties with enhanced disease resistance ultimately laying the foundation for cultivating higher-quality disease-resistant sugarcane.

### 7.2. Precise Resistance Identification Method

The selection of disease-resistant varieties is based on evaluating existing germplasm resources to identify disease-resistant materials and breed new varieties. To quickly and efficiently evaluate these germplasm resources, the best method for assessing disease resistance must be determined. For instance, different resistance identification methods for the same sugarcane variety can yield inconsistent results. For example, variety ROC22 reported high resistance in one study, while another reported medium resistance. Therefore, a unified and systematic study of different resistance identification methods is crucial to find the most accurate method for breeding sugarcane leaf scald-resistant varieties. Currently, there are few reports on the systematic study of sugarcane leaf scald resistance identification methods. The inoculation method, inoculation environment, identification period, disease grading standard, and statistical analysis method have not been unified in the resistance identification process. In summary, there is a lack of unified identification system for resistance. For this reason, it is important to establish a standard identification system for leaf scald in sugarcane. This system should synthesize various indices and unify the standards to create an efficient, rapid, stable, widely adaptable, and precise method for leaf scald resistance identification. This will facilitate research on disease resistance mechanisms and the selection and breeding of disease-resistant varieties. Given the complex genetic background of sugarcane and the lack of immune or highly resistant resources, these issues have led to inefficiencies in selecting disease-resistant varieties through conventional breeding. Future efforts should focus on collecting, identifying, and utilizing foreign disease-resistant germplasm, local domestic germplasm, as well as germplasm from tropical and subtropical regions. Additionally, creating excellent disease-resistant germplasm resources through population improvement, backcrossing, and trans-breeding will broaden the base of disease-resistant germplasm. Establishing an efficient germplasm resource nursery will promote the development of new sugarcane varieties.

### 7.3. Comprehensive Control of Leaf Scald in Sugarcane

To effectively combat leaf scald in sugarcane, future strategies should be focused on augmenting both agricultural and biological control methods. Agricultural control should emphasize the combination of basic theoretical research and production practice. This includes planting disease-resistant varieties, adopting scientific agronomic measures, and constructing a comprehensive control technology system for sugarcane leaf scald based on resistant sugarcane varieties and agronomic measures. In biological control, efforts should focus on exploring and developing more efficient biological agents or on improving the preventive effect of existing biological agents through compounding, adding additives, and other strategies. Additionally, further research on the mechanism of antagonists and field use techniques is required.

## Figures and Tables

**Table 1:** General information about public 21 *Xanthomonas* strains.

Strain	Size (Mb)	BioSample	%GC	Assembly	Scaffolds	CDS	Geographic Location	Collection	Reference
Xa04	3.78504	SAMN24462201	62.98%	Complete	1	3141	Brazil: Sao Paulo	2010	[52]
Xa11	3.90843	SAMN24462202	62.91%	Complete	1	3255	Brazil: Sao Paulo	2010	[52]
Xa21	3.95909	SAMN24462203	62.88%	Complete	1	3283	Brazil: Sao Paulo	2010	[52]
Xa26	3.88559	SAMN24462204	62.97%	Complete	1	3283	Brazil: Sao Paulo	2010	[52]
Xa-FJ1	3.75612	SAMN12905433	62.97%	Complete	2	2968	China	2015	[51]
GPEPC73	3.85230	SAMEA3138291	62.91%	Complete	4	3105	Guadeloupe	2003	[50]
HVO005	3.65265	SAMN03262630	62.90%	Scaffold	2	2912	Burkina Faso	1979	[52]
HVO082	3.63602	SAMN03262511	63.00%	Scaffold	1	2878	Burkina Faso	1989	[52]
CFBP2523	3.68418	SAMN05526486	63.10%	Scaffold	148	2996	Fiji	1961	[52]
FIJ080	3.68125	SAMN03257616	63.00%	Scaffold	1	2929	Fiji	1961	[52]
REU209	3.68727	SAMN03262627	63.00%	Scaffold	2	2921	France	1995	[52]
REU174	3.86323	SAMN03263614	62.80%	Scaffold	29	3097	France	1995	[52]
GAB266	3.79190	SAMN03263609	62.90%	Scaffold	4	3013	Gabon	2011	[52]
GPEPC86	3.85143	SAMN03262651	62.80%	Scaffold	4	3158	Guadeloupe	2003	[52]
GPEPC17	3.81135	SAMN03262650	62.80%	Scaffold	4	3122	Guadeloupe	2003	[52]
MTQ032	3.80155	SAMN03257639	62.90%	Scaffold	4	3079	Martinique	1932	[52]
PNG130	3.54265	SAMN03262494	63.30%	Scaffold	4	2868	Papua New Guinea	1993	[52]
LKA070	3.66795	SAMN03262508	63.10%	Scaffold	2	2940	Sri Lanka	1962	[52]
USA048	3.58204	SAMN03262645	63.10%	Scaffold	6	2828	USA	1986	[52]
Xa23R1	3.54903	SAMN03262642	63.10%	Scaffold	4	2839	USA	1993	[53]
XaFL07-1	3.79895	SAMN03262647	62.90%	Scaffold	6	3111	USA	2007	[52]

**Table 2:** Sugarcane leaf scald-specific primers.

Target Gene	Primer	Forward or Reverse Primer	Sequence (5′-3′)	Product Size/bp	Reference
*ITS*	Ala4/L1	F	CCCGACTGGCTCCACCACTG	360 bp	[56]
		R	CAAGGCATCCACCGT		
*ITS*	PGBL1/PGBL2	F	CTTTGGGTCTGTAGCTCAGG	288 bp	[57]
		R	GCCTCAAGGTCATATTCAGC		
*ABC*	XAF1/XAR1	F	CCTGGTGATGACGCTGGGTT	608 bp	[58]
		R	CGATCAGCGATGCACGCAGT		
albicidin toxin biosynthesis gene	XaQf/XaQr	F	TTTGCGGTGTCGGTAAAGGAG	148 bp	[59]
		R	GCGATGGCACTAGGTACAGC		

**Table 3:** Disease severity of leaf scald in sugarcane.

Grade	Symptom
Score 0	Asymptomatic
Score 1	One or two white pencil lines
Score 2	More than two white pencil lines
Score 3	Chlorotic or yellowing leaf
Score 4	Leaf necrosis
Score 5	Plant death

**Table 4:** Criteria for evaluation of resistance to leaf scald based on disease index in sugarcane.

Resistance Evaluation	Disease Index (%)
High resistant	Disease index ≤ 5.0
Resistant	5.0 < Disease index ≤ 15.0
Medium resistant	15.0 < Disease index ≤ 30.0
Susceptible	30.0 < Disease index ≤ 50.0
High susceptible	Disease index > 50.0

**Table 5:** Resistant sugarcane materials of leaf scald disease in different countries.

No.	Resistance Materials	Source	LSD Resistance	Reference
USA				
1	CP09-2392	Florida	Resistant	[117]
2	CP10-2195	Florida	Resistant	[118]
3	HoCP00-950, L01-283, HoCP04-838	Texas	Resistant	[119]
4	CP70-321, HoCP96-540, L07-57, Ho08-711, Ho08-717, HoCP08-726, L08-88, L08-92	Gabrielle	Resistant	[115]
5	CP05-1526	Florida	Resistant	[120]
6	CP72-2086	Florida	Resistant	[121]
7	L99-233, L03-371	Louisiana	Resistant	[122]
8	L97-128	Florida	Resistant	[123]
9	CP00-1101	Florida	Resistant	[124]
10	L88-63, CP79-318, CP65-357, LHo83-153	Gabrielle	Resistant	[125]
11	LCP85-384	Florida	Resistant	[126]
12	CP06-2425, CP06-2495, CP06-2964, CP06-3103, CP89-2143	Florida	Resistant	[127]
13	CP78-1628	Florida	Medium resistance	[117]
14	L99-226	Texas	Medium resistance	[119]
15	CP09-1952	Florida	Medium resistance	[128]
16	L01-299	Louisiana	Medium resistance	[122]
17	CPCL97-2730	Florida	Medium resistance	[129]
18	CP01-1372	Florida	Medium resistance	[130]
19	CPCL99-4455	Florida	Medium resistance	[131]
20	CP00-2180	Florida	Medium resistance	[129]
21	CP07-1313, CL88-4730	Florida	Medium resistance	[127]
France				
22	R570	Guadeloupe	Resistant	[25]
23	FR95285, FR94129, FR88196	Guadeloupe	Resistant	[42]
Gabon				
24	Co6415	Franksville	High resistance	[132]
25	B8008	Franksville	Resistant	[132]
México				
26	Co997	Veracruz	Resistant	[133]
27	Q96, CP74-2005, RD75-11	Veracruz	Resistant	[134]
Australia				
28	Co740, SP70-1423, Q84, Q90, Q110, Q115, Q117, Q120		High resistance	[113]
29	Q124		Resistant	[113]
Cuba				
30	C1051-73	Jovellanos	Resistant	[12]
China				
31	Zhongzhe9,Zhongzhe2, GUC19, GUC8, Yunrui03-103, Yunrui05-649, Yunrui05-182, Yunrui05-367, Yunrui89-159, ROC22, Funong11601, Funong09-4059, Guitang02-467, Guitang08-297	Guangxi	High resistance	[39]
32	Zhongzhe5, GUC13, GUC9, Yunrui03-394, ROC10, ROC23, ROC25, ROC1, Funong5, Funong07-3206, Guitang05-2605, Guitang00-245, GUC25, GUC35	Guangxi	Resistant	[39]
33	Yuegan50, Funong09-7111, Zhongzhe10	Fujian	Resistant	[111]
34	Zhongzhe13	Fujian	Resistant	[116]
35	NCo310, F156, F160, F170, F173	Taiwan	Resistant	[135]
36	Dezhe12-88, Funong11-2907Yuegan49, Zhongzhe1, Haizhe28, Yuegan51	Guangxi, Guangdong	Medium resistance	[26]
37	Guitang40, Guitang08-120, Liucheng07-150, Yunzhe11-3898, Yuegan53, Mintang11-610, Yunzhe09-1601, Yuegan43	Fujian	Medium resistance	[111]
38	Funong14-1854, Liucheng05-136, Yunzhe15-505	Fujian	Medium resistance	[116]
39	Q42, Q50, Q98, Q813, POJ36, POJ2725, CP807, CP29-116, Co290, Co301, Co331, Co421, B4908	Taiwan	Medium resistance	[135]
40	ROC19	Taiwan	Medium resistance	[136]

## Data Availability

No new data were created or analyzed in this study. Data sharing is not applicable to this article.
